# A Novel U-Shaped Association Between Serum Magnesium on Admission and 28-Day In-hospital All-Cause Mortality in the Pediatric Intensive Care Unit

**DOI:** 10.3389/fnut.2022.747035

**Published:** 2022-02-21

**Authors:** Chao Yan Yue, Chun Yi Zhang, Zhen Ling Huang, Chun Mei Ying

**Affiliations:** ^1^Department of Laboratory Medicine, Obstetrics and Gynecology Hospital of Fudan University, Shanghai, China; ^2^Department of Anesthesiology, Renji Hospital Affiliated to Shanghai Jiaotong University, Shanghai, China

**Keywords:** magnesium, mortality, ICU, pediatric, safe medication

## Abstract

**Objective:**

Our purpose is to evaluate whether serum magnesium when entering the ICU is related to 28-day in-hospital all-cause mortality in the pediatric ICU.

**Methods:**

We used the PIC database to conduct a retrospective analysis to investigate the first-time serum magnesium levels of 10,033 critically ill children admitted to the pediatric ICU, and analyzed association between serum magnesium and all-cause mortality. Smoothing spline plots, subgroup analysis and segmented multivariate logistic regression analysis were conducted to estimate the relative risk between serum magnesium and all-cause mortality. The shape of the curve was used to describe the relationship between magnesium and 28-day in-hospital mortality.

**Results:**

There is a non-linear relationship between serum magnesium and 28-day in-hospital all-cause mortality. The U-type relationship between serum magnesium and all-cause mortality was observed. The optimal range of serum magnesium with the lowest risk of mortality was 0.74–0.93 mmol/L. As the serum magnesium level reaches the turning point (0.74 mmol/L), the risk of death decreases by 60% for every 0.1 mmol/L increase in serum magnesium; when the serum magnesium level exceeds 0.93, an increase of 0.1 mmol/L increases the risk of death by 38 %.

**Conclusion:**

Serum magnesium has a U-shaped relationship with 28-day in-hospital all-cause mortality. Both low and high serum magnesium can increase the risk of death. The best serum magnesium range when the risk of death is the lowest is 0.74–0.93 mmol/L.

## Introduction

Magnesium is the fourth most plentiful mineral in the body and the second most plentiful intracellular cation (1). Sixty-seven percentage of magnesium is found in bone, 31% in cells and 1–2% in extra cellular fluids (2). Magnesium is a key electrolyte for maintaining cell membrane potential, a key co-factor for adenylate cyclase and sodium-potassium-adenine triphosphatase, an essential mineral for hundreds of enzymatic reactions (3), and is involved in more than 300 biochemical reactions in the body with a range of important physiological roles (4). Magnesium plays an important role in electrolyte homeostasis, cell membrane stability and blood pressure regulation (5), energy production, storage and utilization, protein metabolism, inflammation (6), promotion of nerve conduction, insulin metabolism, myocardial contraction, regulation of vascular tone, atherosclerosis and thrombosis, proliferation and migration of vascular smooth muscle cells and endothelial cells, and vascular calcification (7).

To date, there is disagreement about the exact nature of magnesium and ICU mortality. Previous clinical studies have focused on mortality and hypomagnesemia, with the majority of studies focusing on a single type of magnesium abnormal status. Some studies have proposed low magnesium (8–10) or high magnesium status (11–13) as risk factors for mortality in ICU patients, while others have shown no association between low magnesium (14–17) and prognosis in ICU patients, and although hypomagnesemia is associated with mortality, it only occurs after ICU admission (14).

Previous studies only focused on adults. To my knowledge, only one study has investigated hypomagnesemia in pediatric ICU patients (18), but there are no studies on the relationship between serum magnesium levels and prognosis in pediatric ICU patients. In this study, we will investigate the serum magnesium levels of pediatric patients at the time of admission to the ICU, and we aim to assess the relationship between serum magnesium and 28-day in-hospital all-cause mortality in pediatric ICU patients.

## Methods

### Subjects

We performed a retrospective analysis using the Pediatric Specialty Intensive Care (PIC) database, selecting 10,033 critically ill children with comprehensive laboratory test results. PIC (Pediatric Intensive Care) is a large pediatric-specific, single-center, bilingual database containing information related to children in the ICU at the Children's Hospital of ZheJiang University School of Medicine, China, from 2010 to 2018. The PIC database includes vital sign measurements, medications, laboratory measurements, fluid balance, diagnosis codes, length of stay, survival data, and more (19). The laboratory indicators we included are the results of the first inspection after admission to the ICU. Inclusion criteria: The results of magnesium are not missing. Exclusion criteria: Outliers (<1% or >99%) for magnesium results. The range of serum magnesium is between 0.59 and 1.58 mmol/L. ICU category including neonatal intensive care unit (NICU), surgery intensive care unit (SICU), pediatric Intensive Care Unit (PICU), cardiac intensive care unit (CICU). Vasoactive drugs including dopamine hydrochloride injection, dobutamine hydrochloride injection, adrenaline hydrochlaride injection, isoprenaline hydrochloride injection, phcnylephrine hydrochloride injection, norepinephrine bitartrate injection. Magnesium supplement drugs include: 25% magnesium sulfate injection, magnesium sulfate powder, hydrotalcite tablets, potassium aspartate and magnesium aspartate injection.

PIC database is a public database, this project was approved by the Institutional Review Board/Ethical Committee of the Children's Hosptial, Zhejiang University School of Medicine (2019_IRB_052). The requirement for individual patient consent was waived because the project did not impact clinical care, and all protected health information was deidentifie (19). We formally applied for access through the procedures recorded on the PIC website and PhysioNet, and we have signed a data usage agreement. We handle data responsibly and adhere to the principle of cooperative research.

### Statistical Analysis

Data is expressed as mean (SD) or median (Q1-Q3) for continuous variables and percentage (%) for dichotomous variables. A smooth curve fit plot of serum magnesium was created to examine the shape of the relationship between serum magnesium and 28-day in-hospital all-cause mortality. We applied a two-segment linear regression model to test the threshold effect of serum magnesium on mortality based on smoothing plots. A segmented regression model was then used to compare the differences between models I and II by performing log-likelihood ratio tests for the single-linear linear regression model and the two-segment linear model. *p* < 0.05 means Model II is significantly different from Model I, which indicates a non-linear relationship.

An additional turning point for serum magnesium was determined by curve fitting the mortality corresponding to the turning point in the graph, and the range between the two points was considered to be the threshold for low risk of death.

Logistic regression models were used to examine the effect of serum magnesium and other variables on the occurrence of 28-day in-hospital all-cause mortality. Because of the small variation in serum magnesium in the human body, the risk associated with 28-day in-hospital all-cause mortality is reported per 0.1 mmol/L of continuous serum magnesium. Data was analyzed with the use of the statistical packages R (The R Foundation; http://www.r-project.org; version 3.4.3). All *P*-values for statistics were two-tailed, and *P* < 0.05 was regarded as statistically significant.

## Results

### Baseline Characteristics and Laboratory Test Results

**Figure 1:**
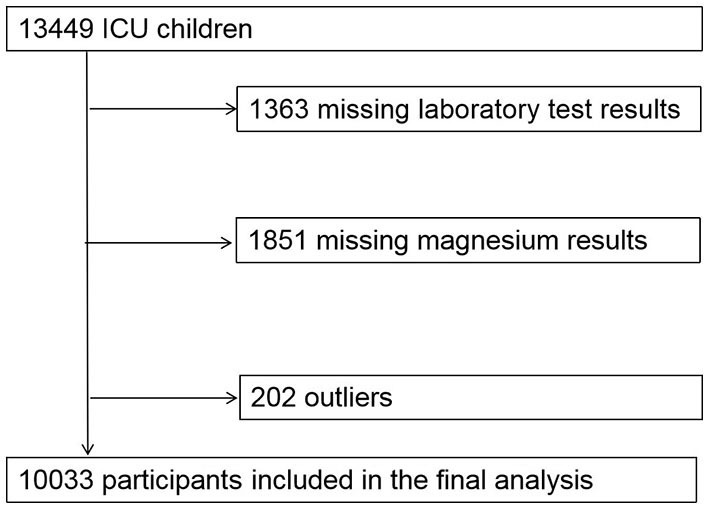
Flowchart of participants.

Figure 1 is flowchart of participants. Our study included 10,033 children, including 5,677 boys and 4,356 girls. Median age at ICU admission was 8.68 months (Q1-Q3: 1.22–40.21), median length of stay in ICU was 1.98 (0.90–7.76) days, median length of stay in hospital was 13.60 (8.02–22.03) days, in-hospital all-cause mortality is 3.83% (384 patients), 28-day in-hospital all-cause mortality is 3.24% (325 patients). The median serum magnesium concentration is 0.87 (0.81–0.94) mmol/L.

Tables 1, 2 describe the baseline characteristics of the subjects, including demographic characteristics and some laboratory test results that may be related to the occurrence of mortality.

**Table 1:** Baseline characteristics of the study participants.

	**28-day in-hospital**	**28-day in-hospital**	***p*** **-value**
	**mortality = 0**	**mortality = 1**	
No. of participants	9,708	325	
Age (months) Median (Q1-Q3)	8.91 (1.32–41.09)	1.78 (0.10–19.00)	<0.001
Vasoactive drugs			0.518
0	7,380 (76.02%)	242 (74.46%)	
1	2,328 (23.98%)	83 (25.54%)	
ICU category, *N* (%)			<0.001
NICU	2,391 (24.63%)	130 (40.00%)	
SICU	2,381 (24.53%)	31 (9.54%)	
CICU	2,413 (24.86%)	38 (11.69%)	
PICU	1,399 (14.41%)	98 (30.15%)	
General ICU	1,124 (11.58%)	28 (8.62%)	
Gender, *N* (%)			0.052
Male	5,476 (56.41%)	201 (61.85%)	
Female	4,232 (43.59%)	124 (38.15%)	
Magnesium supplement, *N* (%)			0.104
0	9,035 (93.07%)	310 (95.38%)	
1	673 (6.93%)	15 (4.62%)	
Prematurity			0.063
0	9,032 (93.04%)	311 (95.69%)	
1	676 (6.96%)	14 (4.31%)	
Length of ICU stay, median (Q1-Q3)	1.96 (0.90–7.68)	3.95 (1.11–8.91)	0.002
Length of hospital stay, median (Q1-Q3)	13.81 (8.10–22.61)	5.14 (1.74–11.84)	<0.001

**Table 2:** Characteristics of clinical laboratory results of study participants.

	**28-day in-hospital**	**28-day in-hospital**	***p*** **-value**
	**mortality = 0**	**mortality = 1**	
No. of participants	9,708	325	
Magnesium (mmol/L)	0.89 ± 0.13	0.95 ± 0.20	<0.001
Albumin (g/L)	39.55 ± 6.77	33.60 ± 8.27	<0.001
Hemoglobin (g/L)	124.24 ± 29.22	125.15 ± 38.99	0.591
Tcho (mmol/L)	3.38 ± 1.28	2.61 ± 1.63	<0.001
ALT (U/L), Median (Q1-Q3)	17.00 (11.00–29.00)	24.00 (14.00–75.00)	<0.001
Creatinine (μmol/L)	72.42 ± 358.56	78.25 ± 62.52	<0.001
Calcium (mmol/L)	2.31 ± 0.24	2.14 ± 0.30	<0.001
Phosphate (mmol/L)	1.86 ± 0.65	2.18 ± 1.03	<0.001
Neutrophils (×10^∧^9/L)	5.63 ± 5.28	8.35 ± 6.96	<0.001
Lactate (mmol/L)	2.38 ± 2.02	5.61 ± 5.34	<0.001
Blood culture results			0.001
0	8,960 (92.30%)	284 (87.38%)	
1	748 (7.70%)	41 (12.62%)	

### Threshold Effect of Magnesium and 28-Day Mortality

The results in Table 3 show that the turning point value (0.74 mmol/L) of magnesium was found through the piece-wise regression model between magnesium and the risk of 28-day in-hospital all-cause mortality. An additional turning point for serum magnesium was determined by curve fitting the mortality corresponding to the turning point in the graph, and the range between the two points (0.74–0.93 mmol/L) was considered to be the threshold for low risk of death.

**Table 3:** Threshold effect analysis for the relationship between magnesium (per 0.1 mmol/L) and 28-day in-hospital all-cause mortality.

**Models**	**Risk of mortality adjusted OR (95%CI)**	***P*** **-value**
**Model I**
One line slope	1.30 (1.19, 1.42)	<0.0001
**Model II**
Turning point (K)	7.4	
<7.4 slope 1	0.41 (0.24, 0.70)	0.0011
> 7.4 slope 2	1.39 (1.27, 1.52)	<0.0001
Slope 2–Slope 1	3.39 (1.92, 5.98)	<0.0001
Predicted at 7.4	−4.20 (−4.39, −4.00)	
LRT test	<0.001	

*Data were presented as OR (95%CI) P-value; Model I, linear analysis; Model II, non-linear analysis. LRT test, Logarithmic likelihood ratio test (p < 0.05 means Model II is significantly different from Model I, which indicates a non-linear relationship); adjust for gender, vasoactive drugs, age, ICU category, magnesium supplement, albumin; hemoglobin; ALT; Creatinine; absolute neutrophil; lactate; Tcho, Calcium, phosphorus, prematurity, blood culture results, length of ICU stay. p < 0.05 indicates that Model II is significant different from Model I*.

The risk of 28-day in-hospital mortality decreased by 59% in patients per 0.1 mmmol/L unit increase of magnesium when magnesium ranged from 0.59 to 0.74 mmol/L and increased by 39% in patients per 0.1 mmmol/L unit increase of magnesium when magnesium ranged from 0.93 to 1.58 mmol/L (log-likelihood ratio test: *P* < 0.001, it demonstrated a non-linear relationship between magnesium and risk of 28-day in-hospital all-cause mortality).

**Figure 2:**
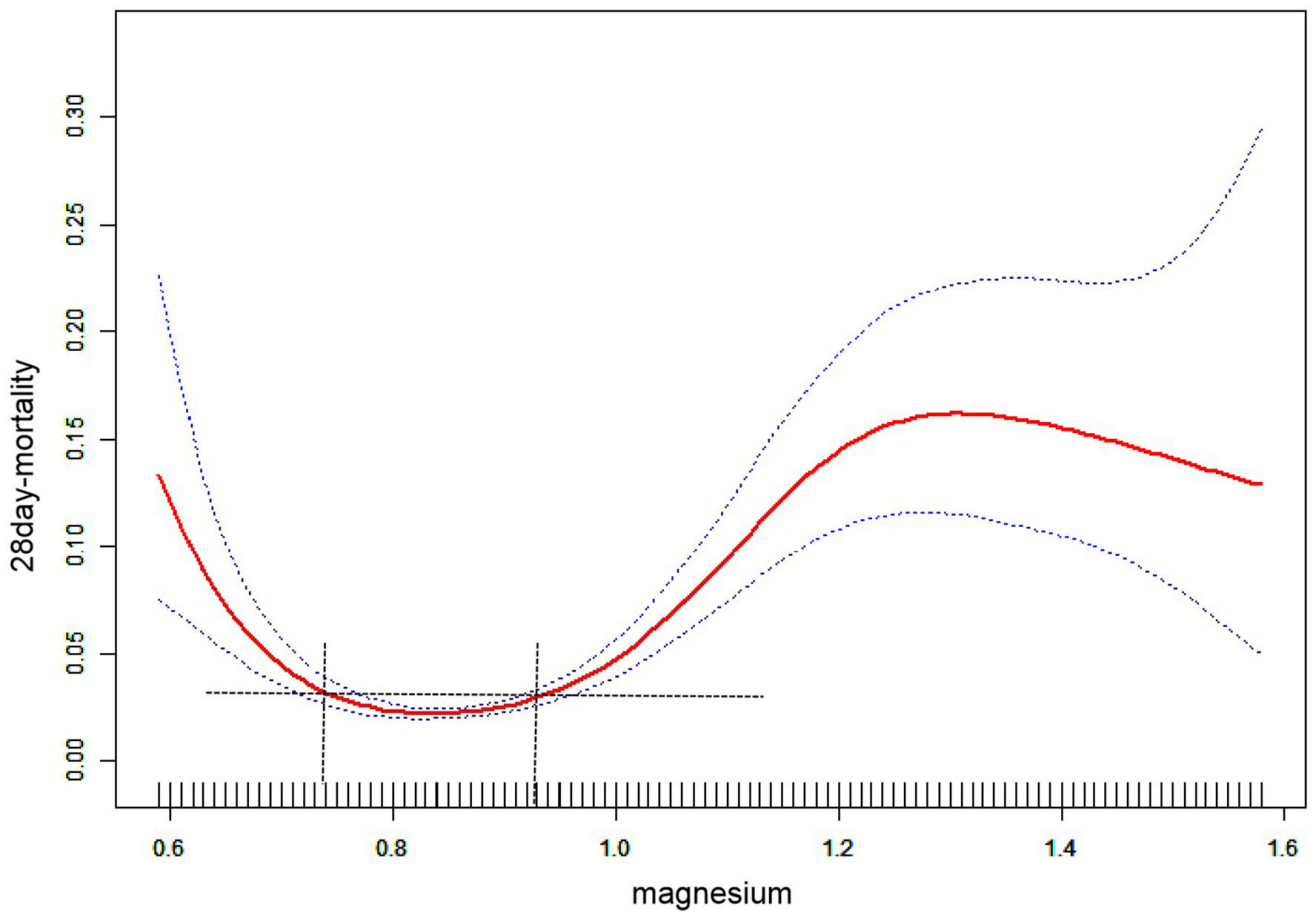
The association between magnesium and 28-day in-hospital all-cause mortality. The red line represents the fitted curve of magnesium and 28-day mortality, and the blue line represents the 95% confidence interval of the curve.

Figure 2 shows a smoothed spline plot of serum magnesium and risk of 28-day in-hospital all-cause mortality. The relationship between serum magnesium and 28-day in-hospital all-cause mortality was U-shaped, with a significant decrease in the incidence of 28-day in-hospital all-cause mortality with increasing serum magnesium concentrations at < 0.74 mmol/L. At >0.93 mmol/L, the incidence of 28-day in-hospital all-cause mortality increased significantly with increasing serum magnesium concentrations.

**Figure 3:**
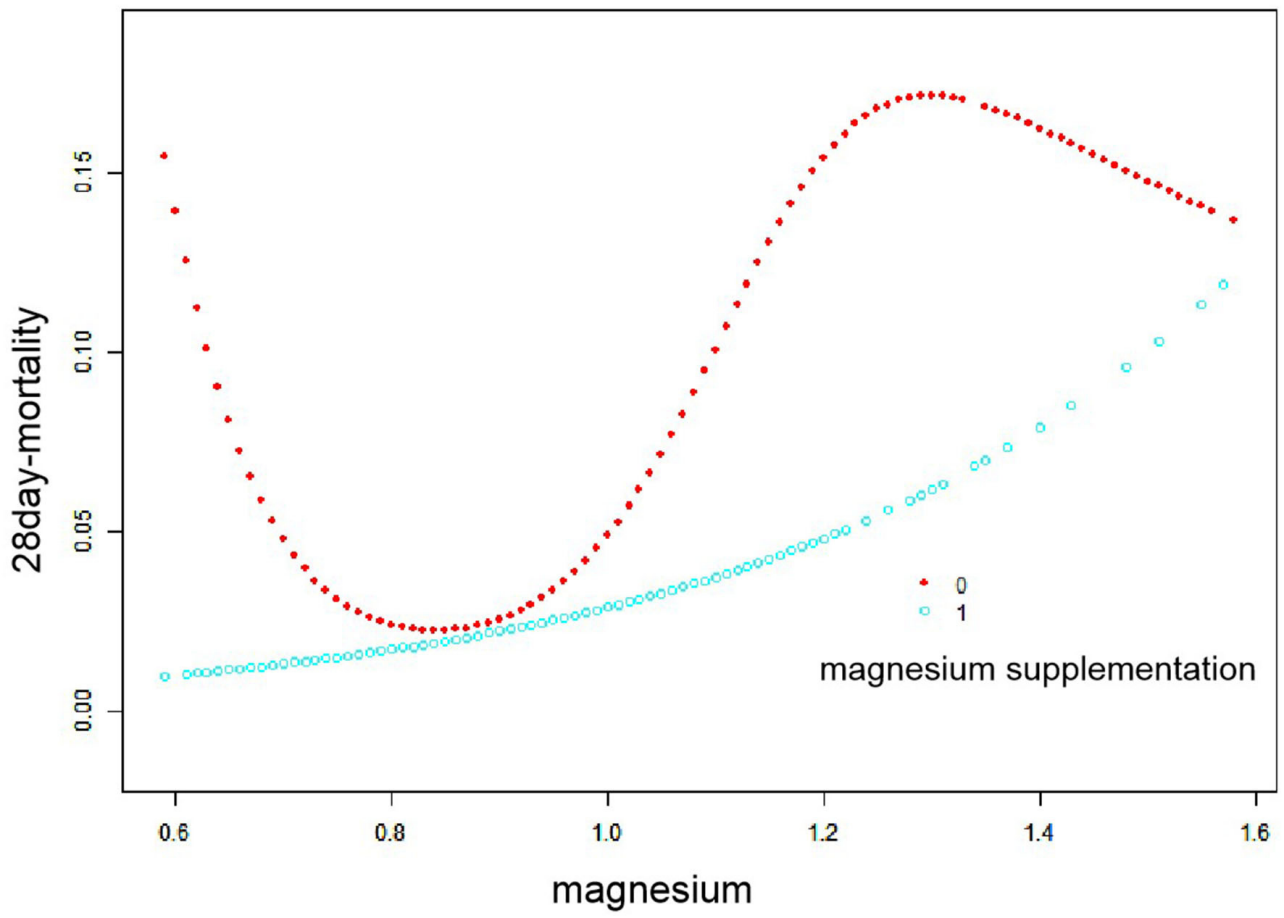
Smooth fitting curve stratified by magnesium supplementation. Red dots represent children who are not taking magnesium supplements, and blue dots represent children who are taking magnesium supplements.

Figure 3 shows a smooth spline plot after stratification according to the use of magnesium supplements or not. In patients without magnesium supplementation, the relationship between serum magnesium and 28-day in-hospital all-cause mortality remained U-shaped. In patients using magnesium supplementation, there was no difference in mortality change with increasing serum magnesium concentrations at <0.74 mmol/L. At >0.93 mmol/L, mortality increased significantly with increasing serum magnesium concentrations.

### Subsection Regression Analysis of Magnesium and 28-Day Mortality

Table 4 shows the results of the multiple regression of the effect of serum magnesium on 28-day in-hospital all-cause mortality. Multivariate regression models included other variables, including sex, age in months, ICU category, vasoactive drugs, magnesium supplementation, albumin; hemoglobin; alanine aminotransfease (ALT); Creatinine; absolute neutrophil; lactate; total cholesterol (Tcho), Calcium, phosphorus, prematurity, blood culture results, length of ICU stay. Serum magnesium of 0.74 and 0.93 mmol/L were used as the cut-off point. When serum magnesium was <0.74 mmol/L, serum magnesium was a protective factor for 28-day in-hospital all-cause mortality. After full adjustment for con-founders, the risk of 28-day in-hospital all-cause mortality was reduced by 59% for each 0.1 mmol/L increase in serum magnesium. When serum magnesium was >0.93 mmol/L, serum magnesium was a risk factor for 28-day in-hospital all-cause mortality. After full adjustment for con-founders, the risk of 28-day in-hospital all-cause mortality increased by 37% for each 0.1 mmol/L increase in serum magnesium.

**Table 4:** Individual effect of magnesium on 28-day in-hospital all-cause mortality using piece-wise linear regression.

**Exposure**	***n***	**Non-adjusted**		**Adjust model I**		**Adjust model II**	
		**OR (95% CI)**	***P*** **-value**	**OR (95% CI)**	***P*** **-value**	**OR (95% CI)**	***P*** **-value**
Magnesium: [0.59–0.74] mmol/L					
Per 0.1 mmol/L	858	0.29 (0.14, 0.59)	0.0006	0.29 (0.14, 0.60)	0.0008	0.41 (0.17, 0.96)	0.0410
Magnesium: (0.93–1.58] mmol/L					
Per 0.1 mmol/L	2,717	1.38 (1.26, 1.52)	<0.0001	1.36 (1.23, 1.50)	<0.0001	1.37 (1.19, 1.57)	<0.0001

*Adjust model I: Adjusted for gender, age, ICU category*.

*Adjust model II: adjust for gender, vasoactive drugs, age, ICU category, magnesium supplement, albumin; hemoglobin; ALT; Creatinine; absolute neutrophil; lactate; Tcho, Calcium, phosphorus, prematurity, blood culture results, length of ICU stay*.

## Discussion

Electrolyte abnormalities are common armong patients in the ICU (20). No comprehensive and uniform understanding of the relationship between serum magnesium and mortality. Our study found a U-shaped relationship between serum magnesium and 28-day in-hospital mortality in the pediatric ICU. There is a non-linear relationship between serum magnesium and mortality, and the threshold effect of serum magnesium on mortality is significant. Before the turning point (0.74 mmol/L), the mortality rate decreased with the increase of serum magnesium level; after the turning point (0.93 mmol/L), the mortality rate increased with the increase of serum magnesium level. To my knowledge, we reveal for the first time a U-shaped relationship between serum magnesium and 28-day in-hospital mortality, presenting strong evidence for a safe range of serum magnesium in magnesium replacement therapy for pediatric ICU patients.

Magnesium deficiency usually occurs in critical illness and is associated with higher mortality and poorer clinical outcomes in the ICU (8–10). The literature reports that hypomagnesemia occurs in 40% of hospitalized patients, ~60% of postoperative patients, 65% of medical ICU patients, and up to 90% of surgical ICU patients (20). Although the vast majority of studies concluded that hypomagnesemia is a risk factor for poor prognosis, there are still several publications reporting no association between hypomagnesemia and prognosis of ICU patients (14–17). Our findings show that in the pediatric ICU, hypomagnesemic status leads to increased mortality when serum magnesium is <0.74 mmol/L and that magnesium supplementation reduces 28-day in-hospital all-cause mortality when patients are first admittion to the ICU unit with serum magnesium <0.74 mmol/L.

Magnesium is an essential co-factor in hundreds of enzyme systems (8). The effects of magnesium on these enzymes, as well as on other important biological processes such as glycolysis, oxidative phosphorylation, nucleotide metabolism, protein biosynthesis, and phosphatidylinositol turnover, emphasize the importance of magnesium in cellular metabolism. Lower magnesium levels are associated with increased interleukin-1, tumor necrosis factor-α, and interferon-γ, are associated with chronic inflammatory stress, and have an impact on mortality (20–23). Magnesium ions regulate intracellular calcium levels, which in turn affect smooth muscle tone. By regulating smooth muscle tone, magnesium deficiency is thought to lead to hypertension, neuromuscular hyperexcitability, bronchial airway constriction, coronary vasospasm, seizures and increased mortality. Disturbed magnesium levels measured in critically ill patients are more common than any other electrolyte (24). However, excessive serum magnesium can cause damage to cardiac systole and diastole, can impair acetylcholine release and reduce muscle sensitivity to acetylcholine. High serum magnesium may lead to severe arrhythmias, myocardial depression, and vasodilation, which can lead to hypotension (25).

There are also conflicting views on the clinical effects of magnesium supplementation. Some studies have proposed that low dietary magnesium intake increases the risk of cardiovascular disease and that magnesium supplementation is beneficial in the treatment of acute myocardial infarction (25). Magnesium supplementation improved blood pressure control (26), insulin sensitivity (27), and endothelial function (28). Magnesium has a protective effect against endothelial cell injury and oxidative stress (29). Prophylactic intravenous magnesium reduces the incidence of postoperative arrhythmias in pediatric patients. Numerous studies have been conducted to address the negative effects of the high prevalence of hypomagnesemia in ICU patients, such as increased mortality, the need for prolonged mechanical ventilation (MV), and increased length of stay in the ICU. The effectiveness of magnesium replacement therapy has been evaluated in several large clinical studies for myocardial infarction, but their results have been inconsistent (30). Similarly studies have concluded that even with the negative effects of hypomagnesemia, there is insufficient evidence for the benefit of magnesium replacement therapy in ICU patients with hypomagnesemia (31). Another study looked at the use of intraoperative magnesium in congenital heart disease in relation to reducing the odds of all postoperative arrhythmias. The results stated that there was no evidence that greater doses of magnesium were associated with a greater reduction in arrhythmia risk, and there was little evidence of a dose-response effect (32). Across the United States, there has been a significant increase in the use of intravenous magnesium for the treatment of children with worsening asthma. However, magnesium replacement therapy has not been associated with changes in hospitalization rates, ICU admissions, or 7-day all-cause readmission rates (33).

With the increased emphasis on avoiding or treating hypomagnesemia, clinicians may not be aware of the potential harm associated with hypomagnesemia, such as hypotension, prolonged QRS time frame, respiratory failure, and even cardiac arrest (24). For example, in a large study of more than 10,000 ICU patients, hypomagnesemia was associated with lower systolic blood pressure and also increased the likelihood of requiring blood pressure-independent vasopressant therapy (34). In a cross-sectional online survey of two national associations of pediatric emergency physicians in Canada and the United States, 24% of respondents taking magnesium had experienced associated severe hypotension requiring treatment and 2% had experienced apnea-related symptoms (35).

In fact, a growing number of clinical studies question the benefits of magnesium supplementation and the safe range of serum magnesium in patients with acute myocardial infarction. A study suggested that the optimal magnesium range for patients with acute myocardial infarction should be lower than the range recommended by current acute myocardial infarction guidelines (36). In addition, the ISIS-4 trial (Fourth International Infarct Survival Study) included 58,050 patients with suspected acute myocardial infarction, but magnesium supplementation did not show a positive effect (37).

Our findings could well explain the above inconsistency regarding the clinical efficacy of magnesium supplementation. It is because of the unique U-shaped relationship between serum magnesium and mortality that simple thinking should not be used to avoid low or high magnesium, but rather to introduce the concept of an optimal magnesium concentration range, which we found to be around 0.74–0.93 mmol/L in pediatric ICU patients, and clinical magnesium supplementation should be done with caution and care and not overkill.

The serum magnesium level is kept constant within very narrow limits. Regulation takes place mainly *via* kidneys. The Mayo Clinic recommends the following serum magnesium reference ranges: 0–2 years: 0.67–1.125 mmol, 3–5 years: 0.67–1.08 mmolL, 6–8 years: 0.67–1.04 mmol, 9–11 years: 0.67–1 mmol, 12–17 years: 0.67–0.96 mmol, >17 years: 0.71–0.96 mmol. In China, the recommended reference range for serum magnesium in the National Clinical Laboratory Procedure is 0.75–1.02 mmol/L for adults and 0.5–0.9 mmol/L for children. One millimoles per litre = 2.4 mg/dl. Due to the influence of age on the concentration of serum magnesium, we adjusted for age as a confounding factor.

One study reported pediatric serum magnesium concentration reference intervals are about 0.55–1.03 mmol/L (38). This reference range is closest to the serum magnesium reference range for Chinese children. However, the normal range suggested in another study for preterm and full-term infants in the first 2 weeks of life was taken as 0.7–1.5 mmol/L (39). In our present study, the median serum magnesium concentration is 0.87 mmol/L, the 25th percentile is 0.81 mmol/L and the 75th percentile is 0.94 mmol/L. The turning point for the threshold effect of serum magnesium on 28-day in-hospital all-cause mortality in the pediatric ICU was 0.74–0.93 mmol/L.

There are some shortcomings in our study; firstly, *in vivo*, magnesium is found almost exclusively in cells. Serum contains only 0.3% of total body magnesium, and total serum magnesium concentrations do not adequately reflect the body's magnesium stores. Because magnesium is a tightly controlled element, serum levels may not reflect magnesium deficiency until the body is severely depleted of magnesium. Magnesium tolerance tests and ionized magnesium ions are alternative laboratory assessment methods; however, each has its own difficulties in the ICU setting (9). Next, this study was a single-center retrospective study, and although every effort was made to adjust for potential confounding factors through multifactorial logistic analysis, there are still other inpatient variables that may confound the predictive effect of serum magnesium, e.g., mechanical ventilation.

## Conclusion

The first determination of serum magnesium in critically ill children in China's ICU has a U-shaped relationship with 28-day in-hospital all-cause mortality. Both low and high serum magnesium can increase the risk of death. The best serum magnesium value when the risk of death is the lowest is 0.74–0.93 mmol/L. In the present study, we present the importance of serum magnesium management in pediatric ICU patients. Future 28-day in-hospital all-cause mortality in pediatric ICU patients could potentially be reduced by aggressive interventions for abnormal magnesium concentrations. Our current work provides data suggesting that serum magnesium levels not only provide important prognostic information about pediatric ICU patients, but that targeting optimal serum magnesium levels may be a promising therapeutic target for magnesium replacement therapy.

## Data Availability Statement

Publicly available datasets were analyzed in this study. This data can be found at: http://pic.nbscn.org//.

## Author Contributions

CY analyzed the data, drafted the manuscript, contributed to study design, and revised the article. CZ contributed to data collation. ZH and CY made contribution to the conception, design, and revision of the revised manuscript. All authors have read and approved the final manuscript.

## Funding

This work was supported by the program for National Natural Science Foundation of China (No. 81902131) and Shanghai Rising Stars of Medical Talents Youth Development Program (SHWRS(2020)_087). Funding sources played no role in the design of the study and collection, analysis or interpretation of data, and played no role in the drafting, revision, or submission of the manuscript.

## Conflict of Interest

The authors declare that the research was conducted in the absence of any commercial or financial relationships that could be construed as a potential conflict of interest.

## Publisher's Note

All claims expressed in this article are solely those of the authors and do not necessarily represent those of their affiliated organizations, or those of the publisher, the editors and the reviewers. Any product that may be evaluated in this article, or claim that may be made by its manufacturer, is not guaranteed or endorsed by the publisher.
